# Supplementary material for: Neurotranscriptomic profiling of deformed wing virus-infected honey bee foragers (Apis mellifera) with different cognitive abilities
Source: Biol Open. 2025 Dec 2;14(11):bio062204. doi: 10.1242/bio.062204 (PMC12714137; doi:10.1242/bio.062204)
Supplement: Supplementary information [file biolopen-14-062204-s1.pdf]

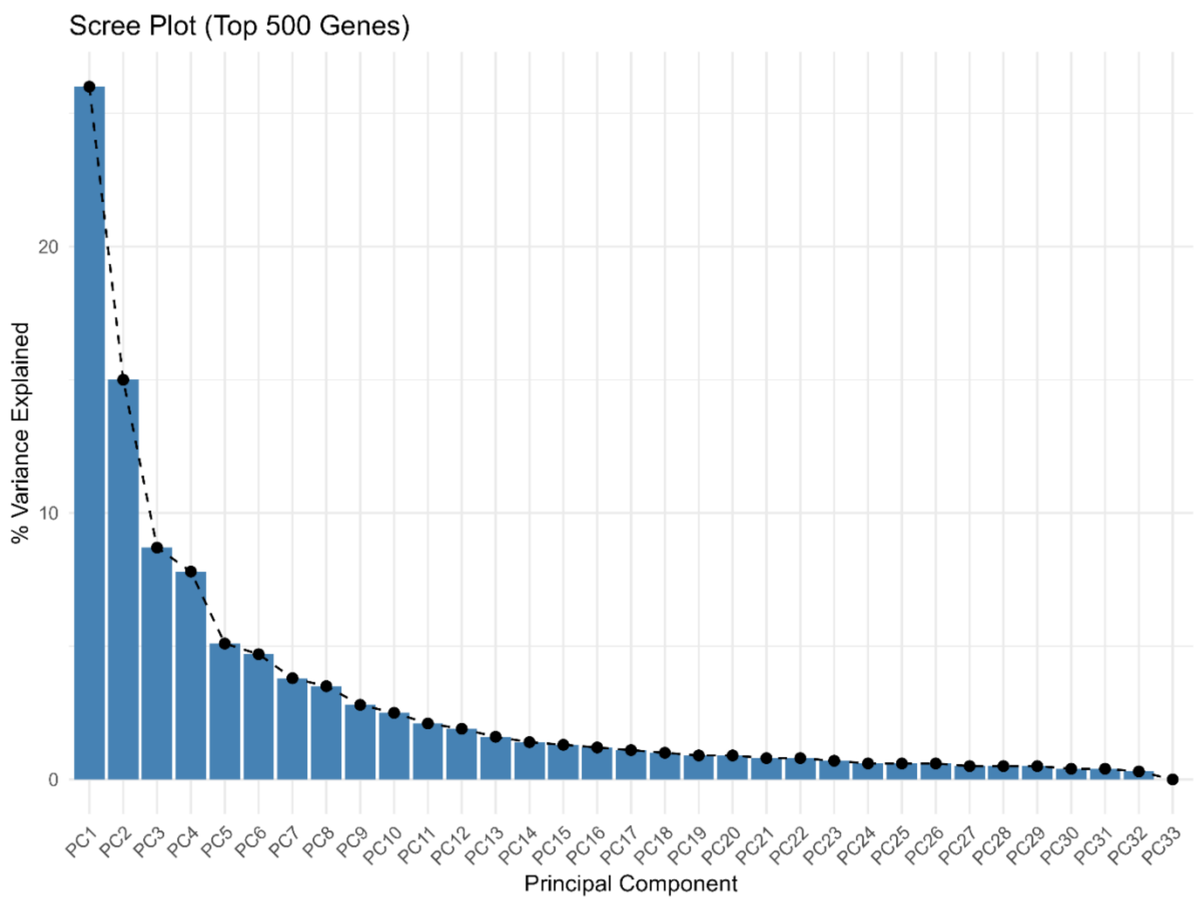

Fig. S1. Scree plot showing the variance explained by each principal component

Biology Open • Supplementary information

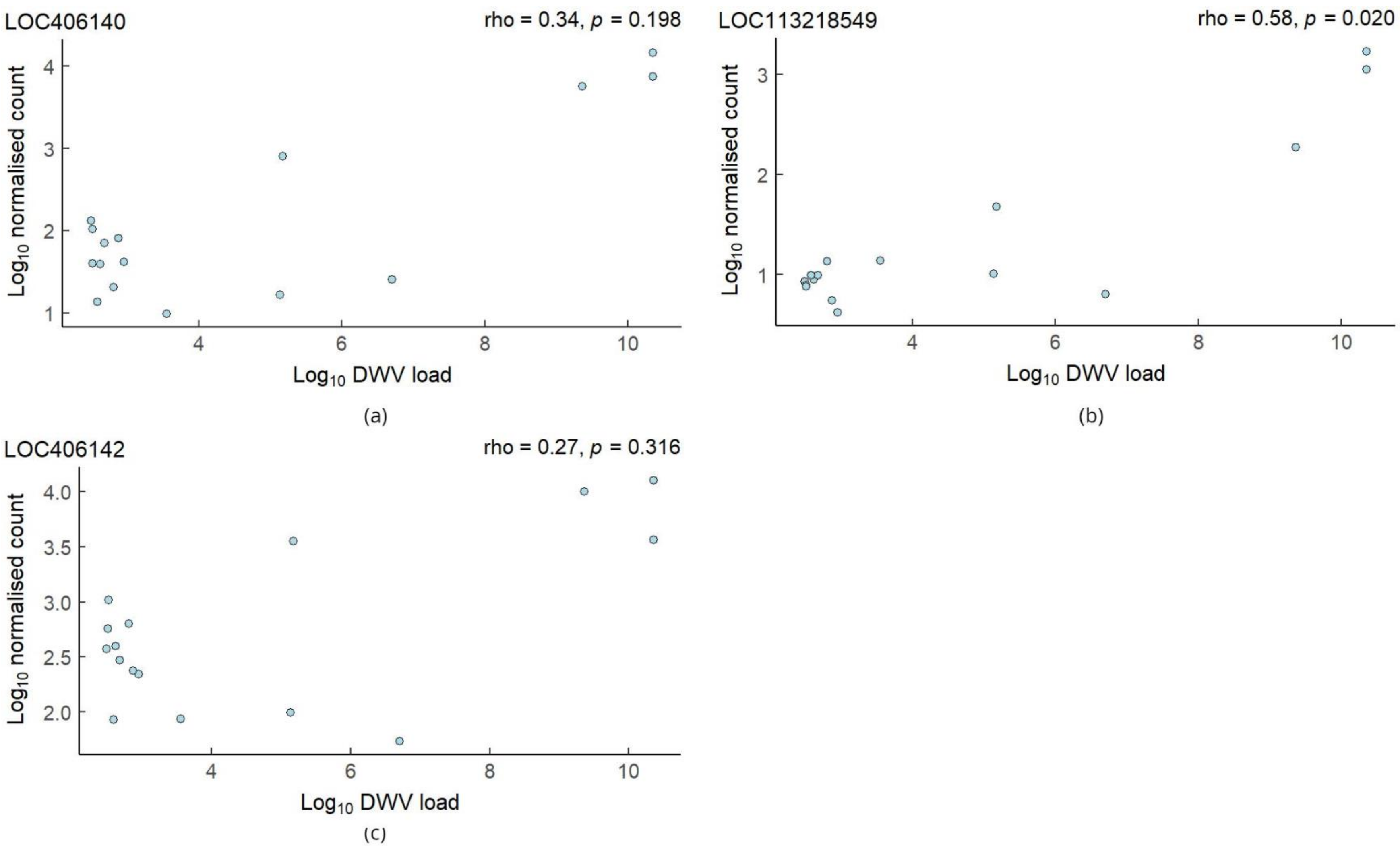

Fig. S2. Correlations between gene counts of upregulated genes and mushroom body viral loads for bees categorised as Poor Learners. (a) LOC406140, *Apid1* (b) LOC113218549 (c) LOC406142, *hymenoptaecin*

Biology Open • Supplementary information

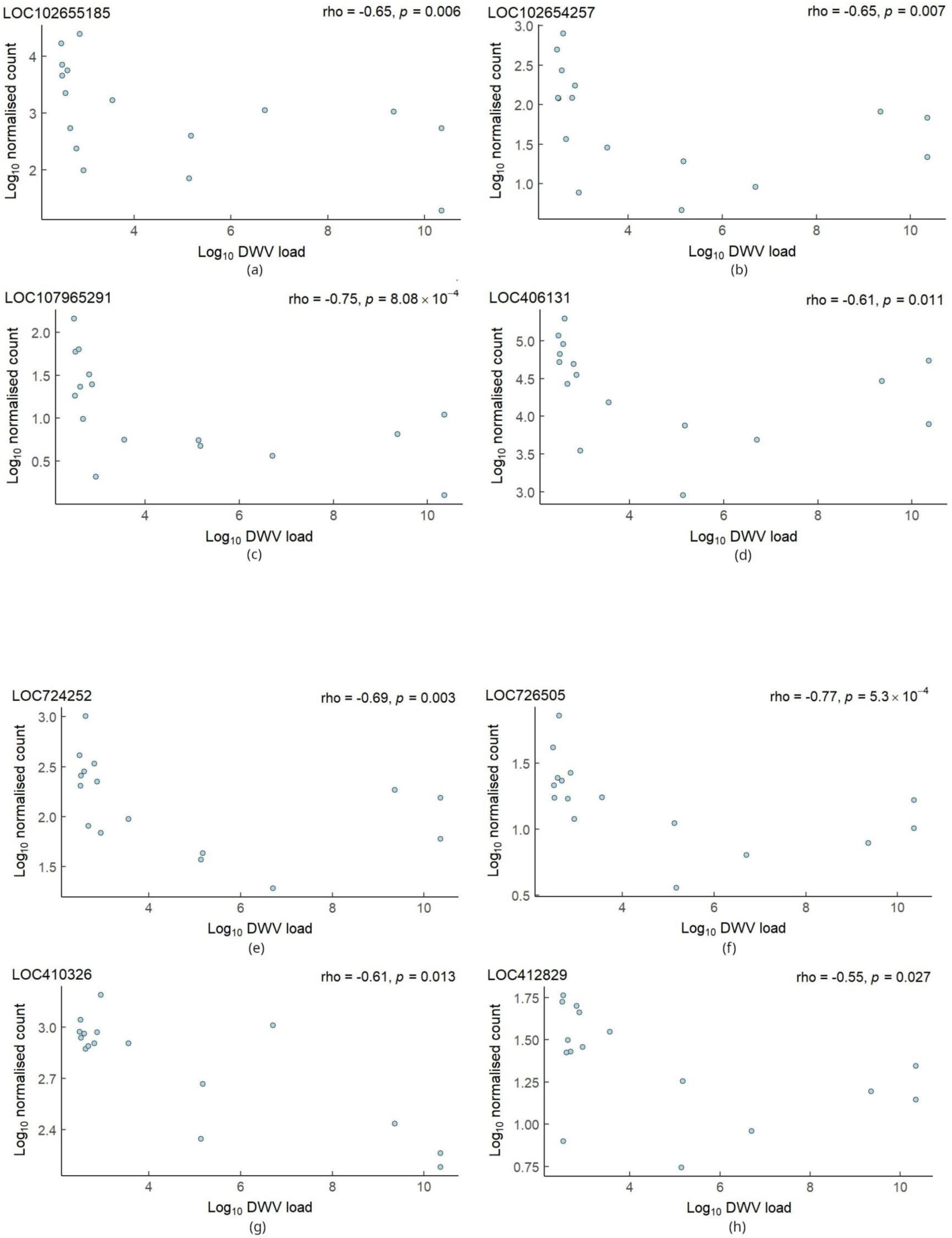

Biology Open • Supplementary information

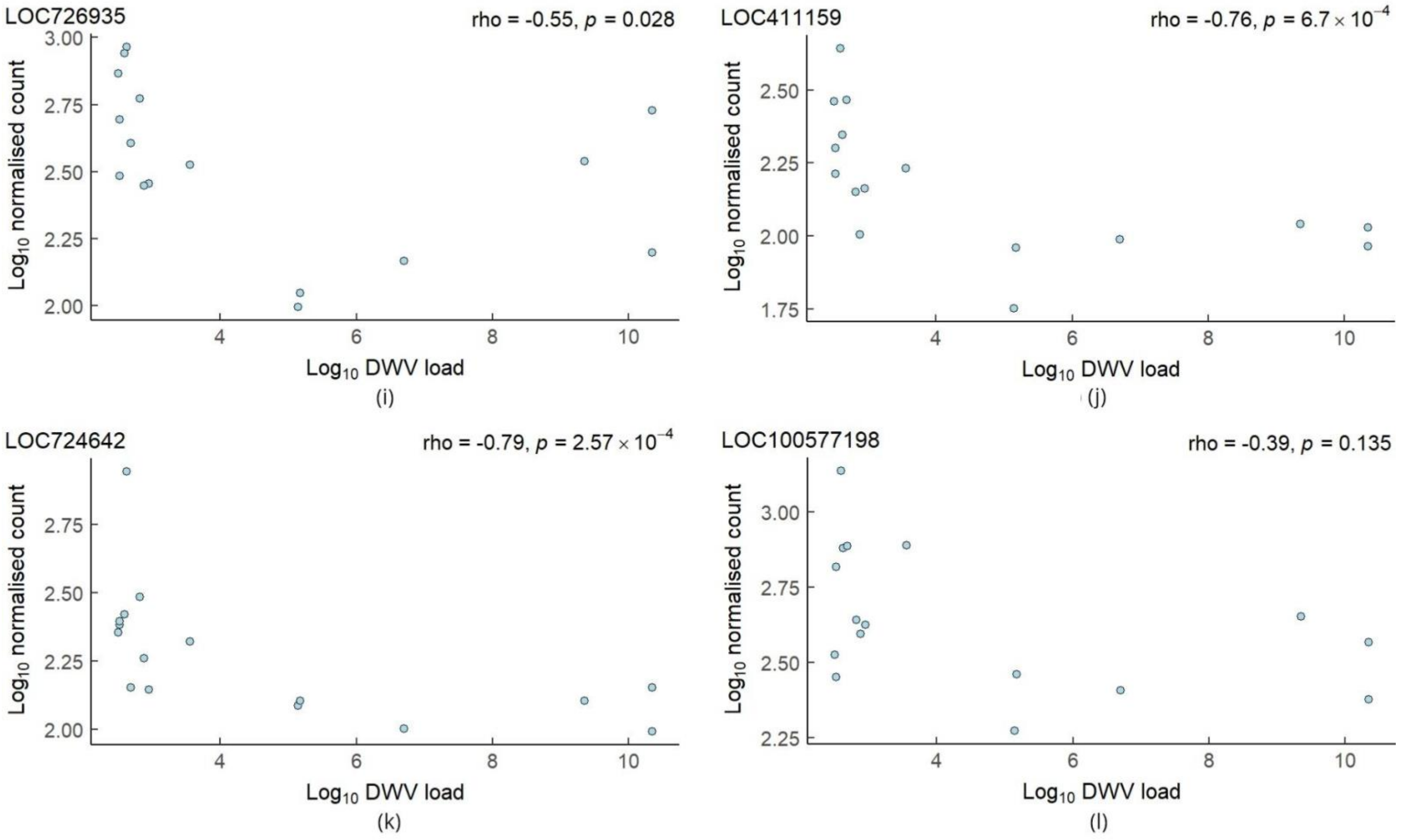

Fig. S3. Correlations between gene counts of downregulated genes and mushroom body viral loads for bees categorised as Poor Learners. (a) LOC102655185 (b) LOC102654257 (c) LOC107965291 (d) LOC406131, *Hbg3* (e) LOC724252, *Dfd* (f) LOC726505 (g) LOC410326 (h) LOC412829 (i) LOC726935 (j) LOC411159 (k) LOC724642 (l) LOC100577198

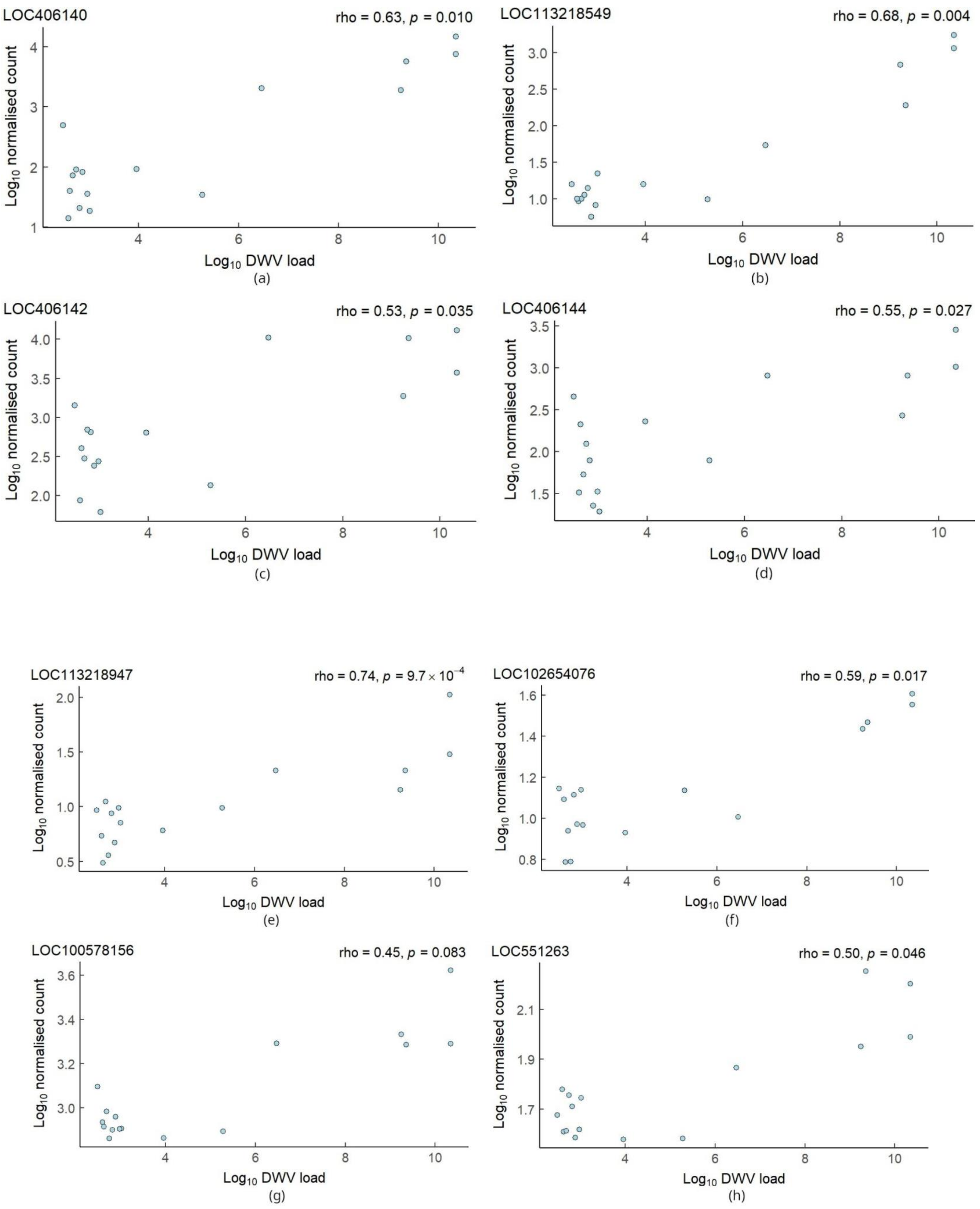

Fig. S4. Correlations between gene counts of upregulated genes and mushroom body viral loads for bees from Colony A. (a) 406140, *Apid1* (b) LOC113218549 (c) LOC406142 (d) LOC406144 (e) LOC113218947 (f) LOC102654076 (g) LOC100578156 (h) LOC551263

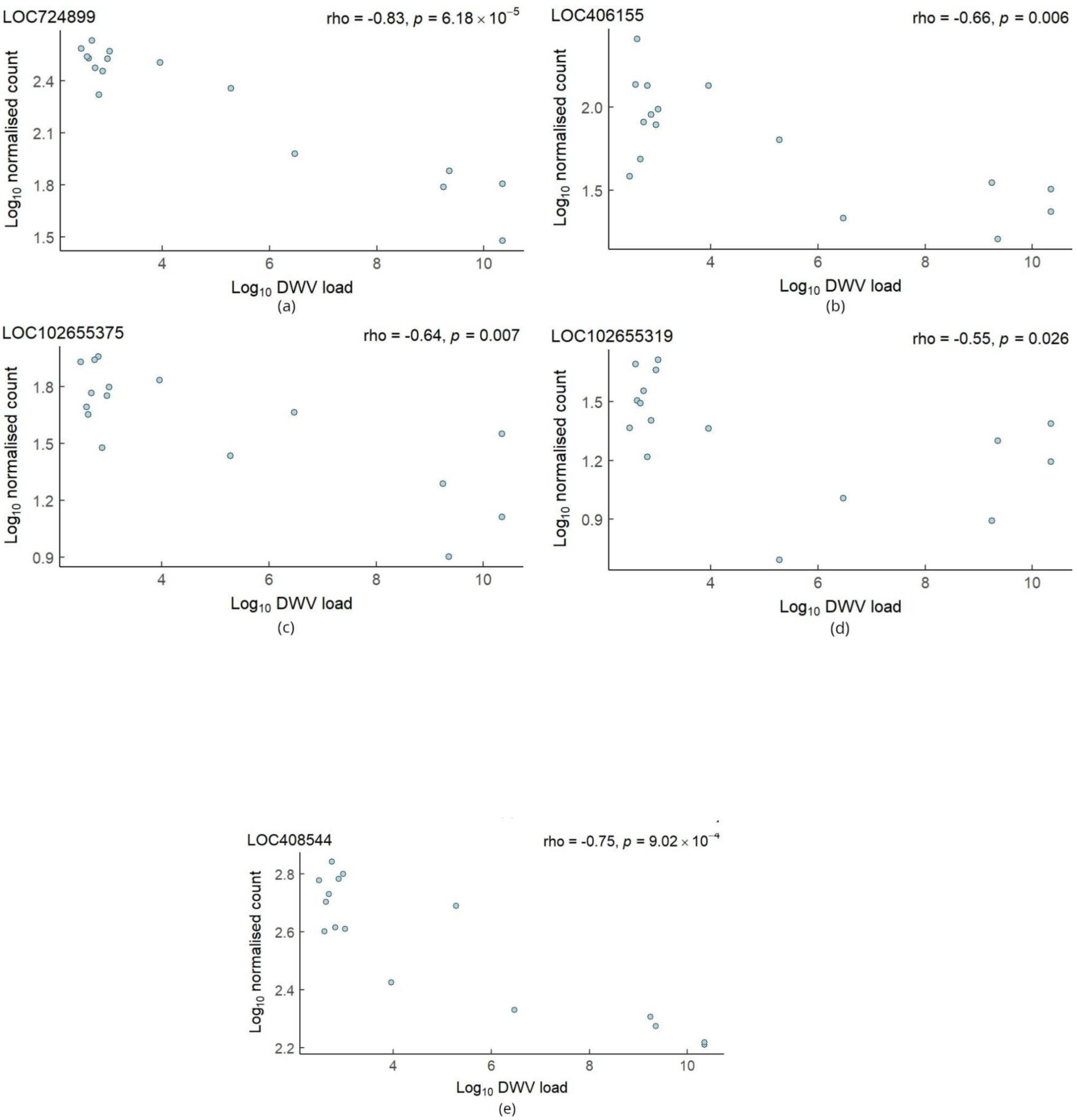

Fig. S5. Correlations between gene counts of downregulated genes and mushroom body viral loads for bees from Colony A. (a) LOC724899 (b) LOC406155, *PPO* (c) LOC102655375 (d) LOC102655319 (e) LOC408544

Newburgh behaviour PC1 and PC2

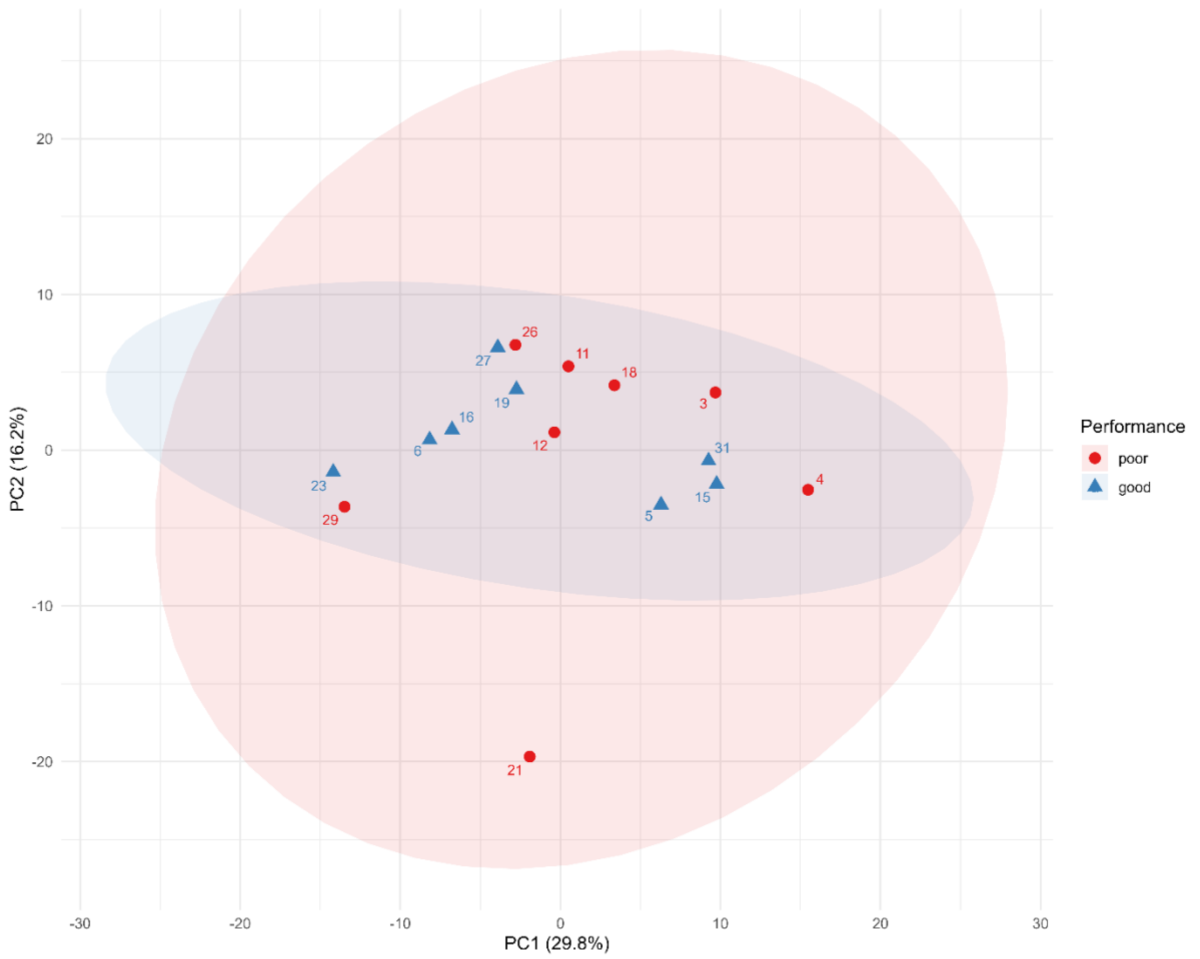

Newburgh behaviour PC2 and PC3

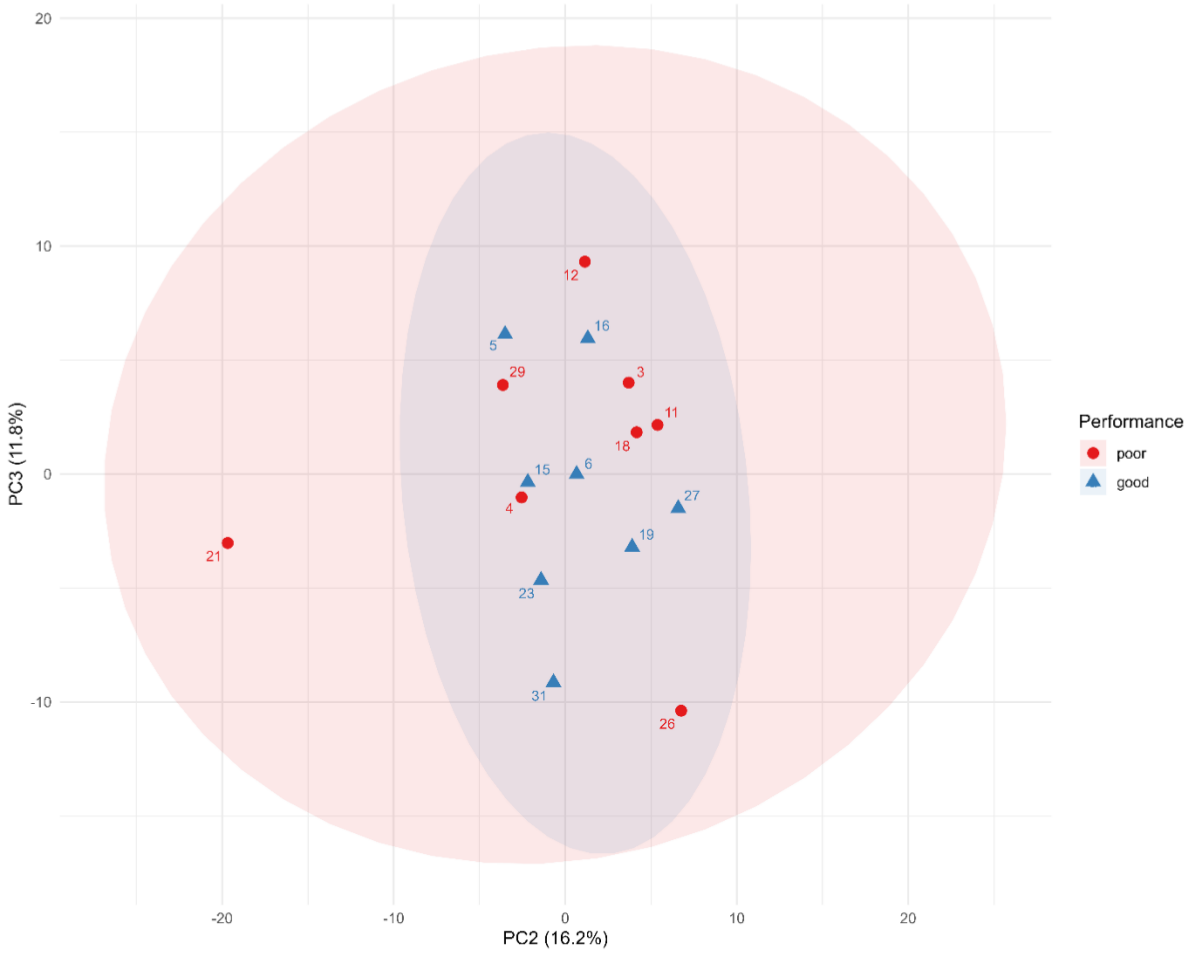

Biology Open • Supplementary information

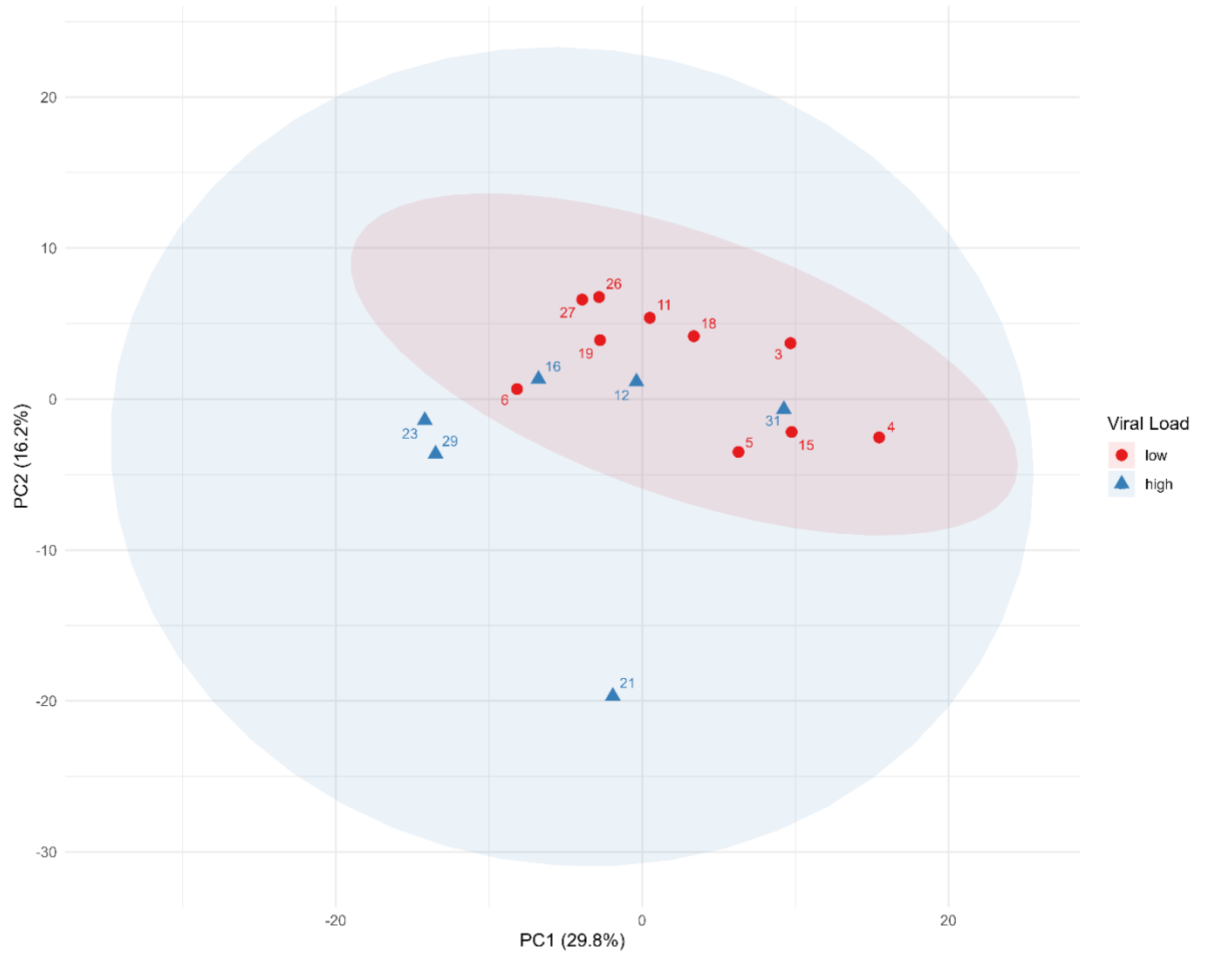

Newburgh viral load PC2 and PC3

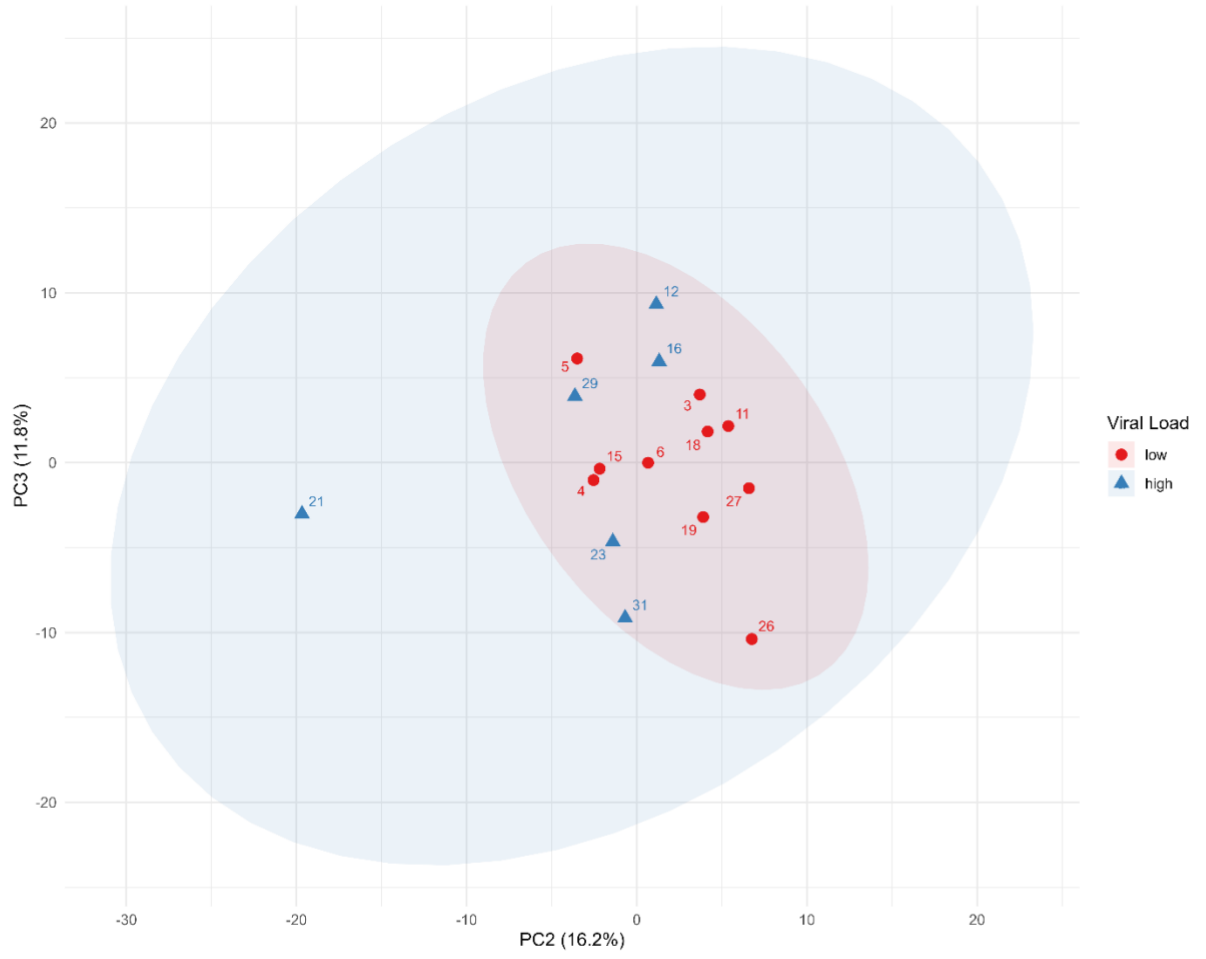

Biology Open • Supplementary information

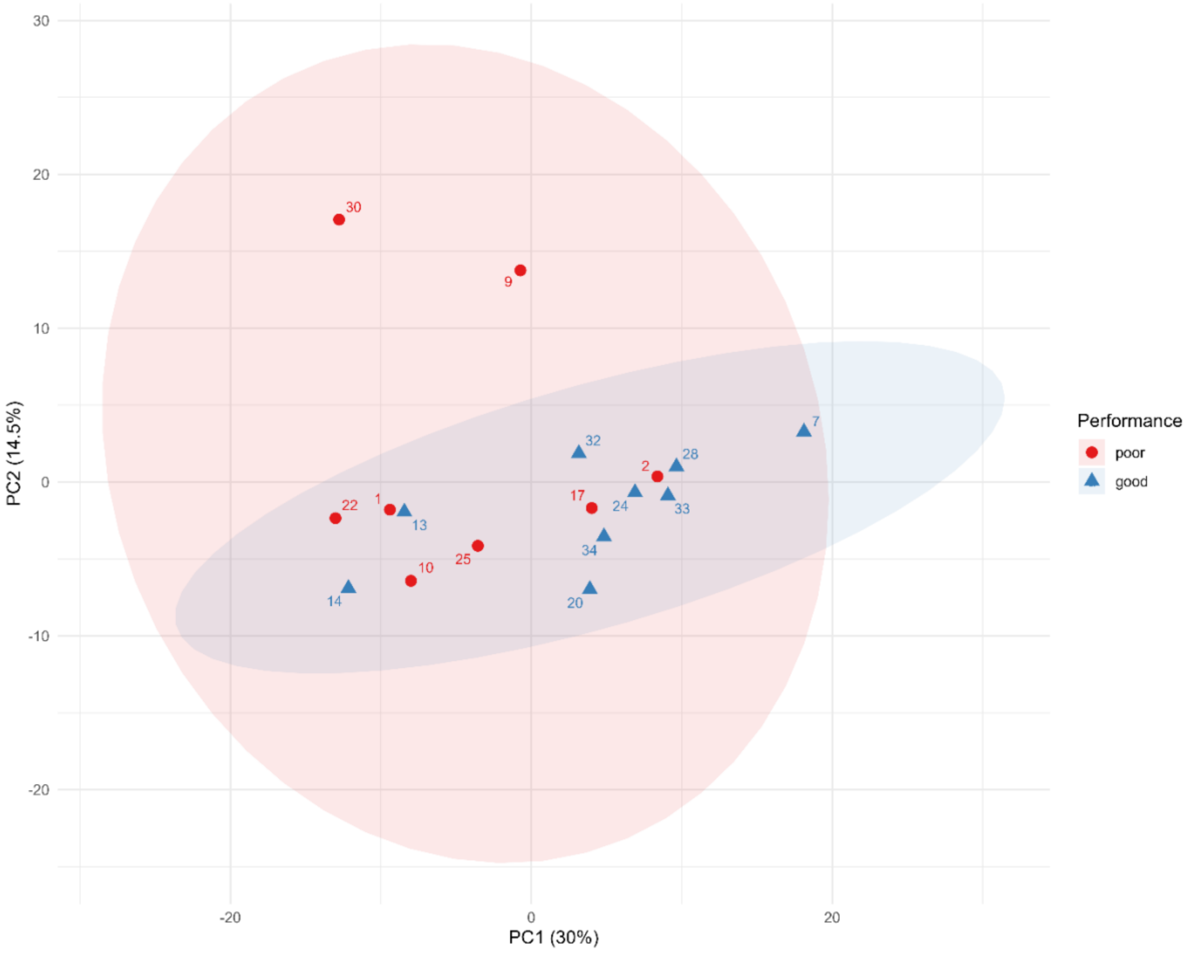

Cruickshank behaviour PC2 and PC3

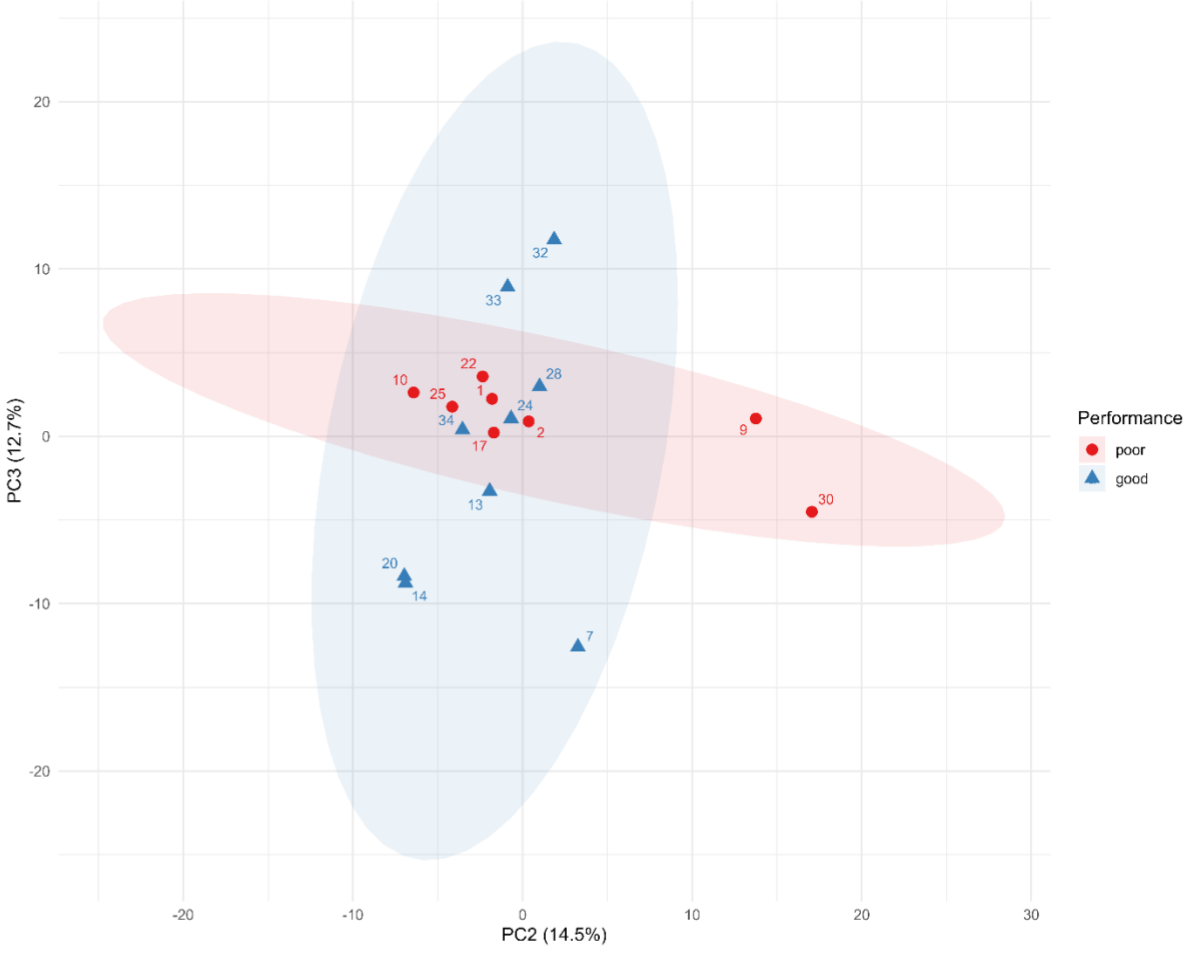

Biology Open • Supplementary information

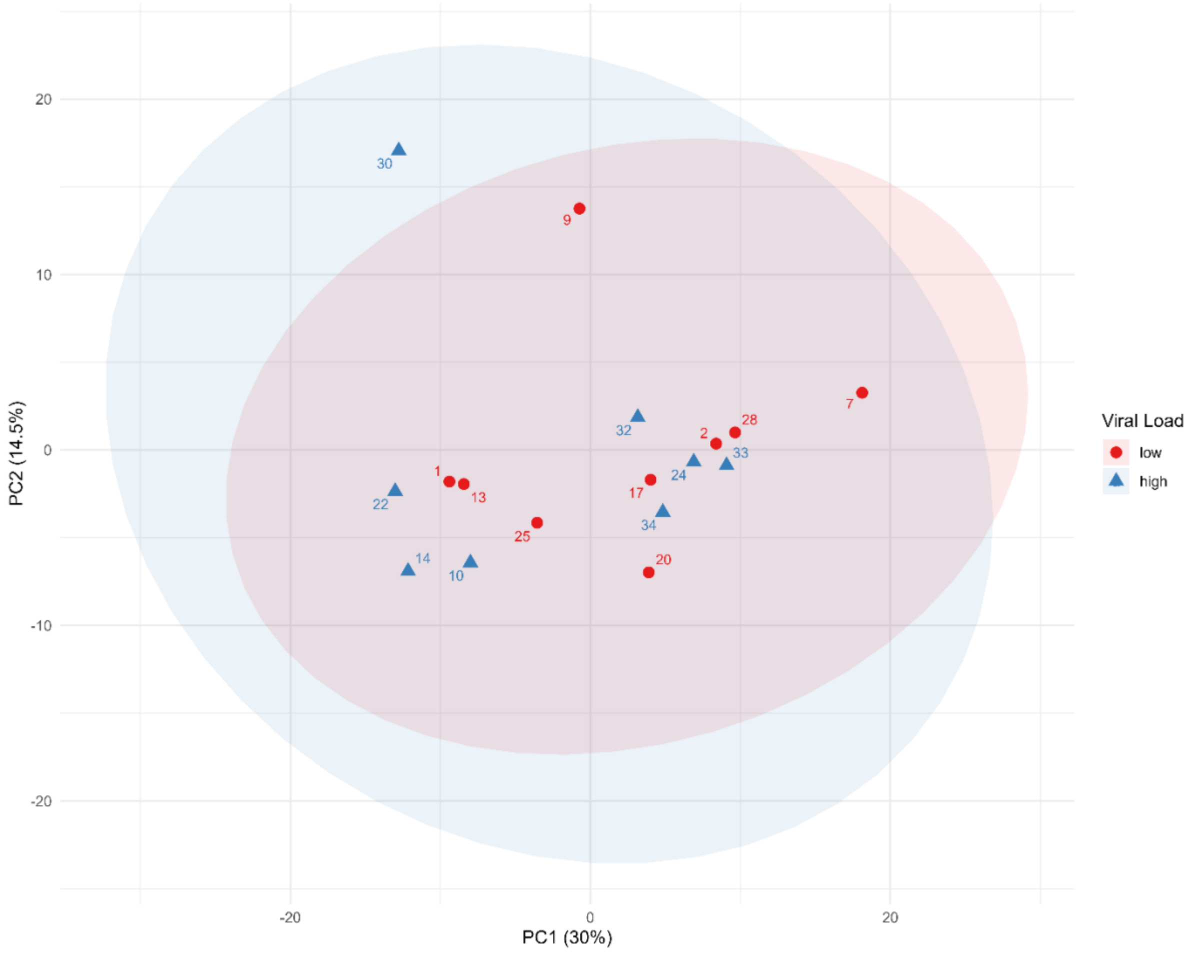

Cruickshank viral load PC2 and PC3

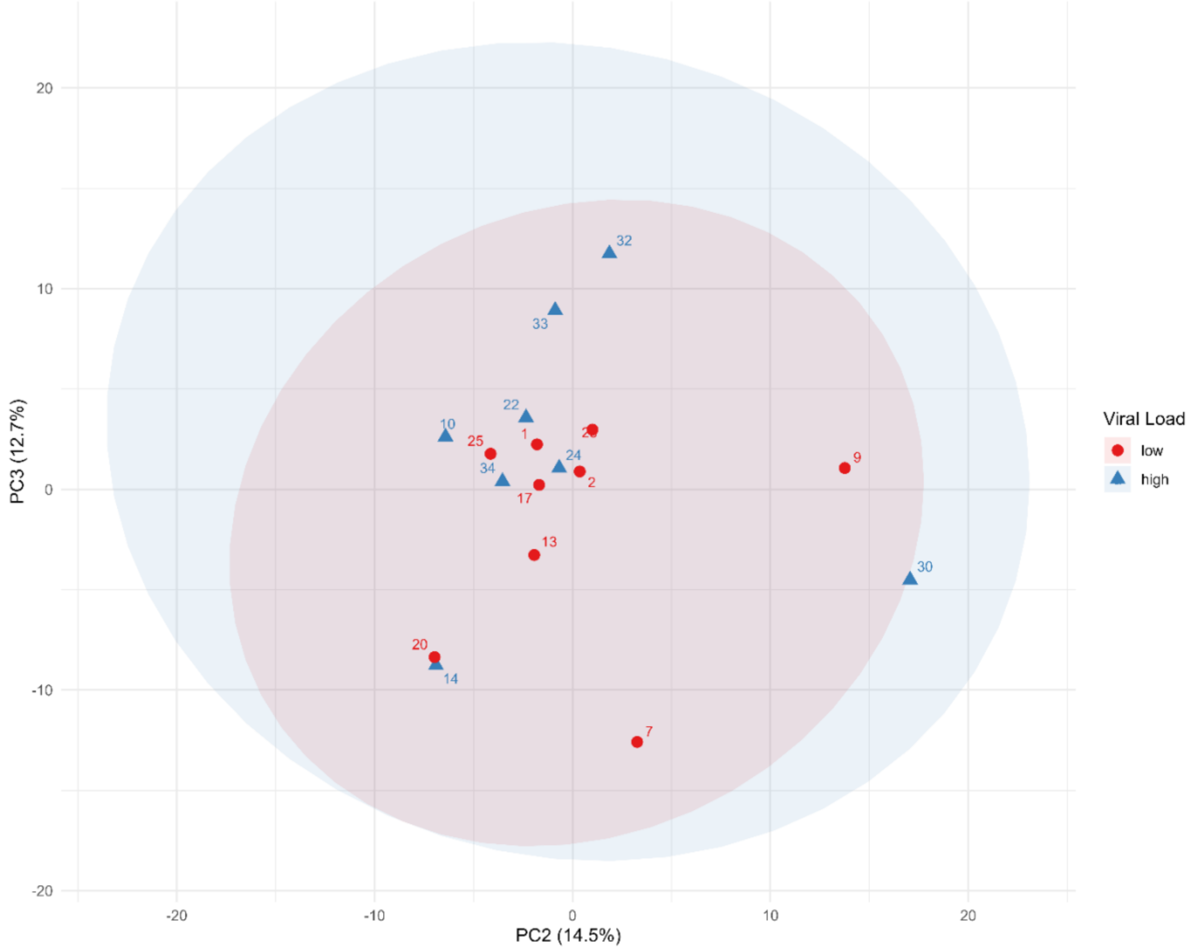

Fig. S6. Output of additional PCA analyses done on the two colonies separately.

Biology Open • Supplementary information

Table S1. KEGG pathways identified via overrepresentation analysis of Poor Learners on 10th November 2025. None of the pathways was significant following a Bonferroni-Hochberg adjustment.

Regulation [1]	KEGG ID	KEGG Description	Genes in Category [2]	Differentially Expressed Genes [3]
Up	ame04745	Phototransduction	9	2
	ame01200	Carbon metabolism	42	2
	ame03040	Spliceosome	49	2
	ame00830	Retinol metabolism	8	1
Down	ame00500	Starch and sucrose metabolism	8	2
	ame00981	Insect hormone biosynthesis	11	2
	ame00030	Pentose phosphate pathway	13	2
	ame01100	Metabolic pathways	417	8
	ame00531	Glycosaminoglycan degradation	5	1

[1] Up = more highly expressed in Poor Learners with high viral loads; Down = lower expression in Poor Learners with high viral loads. [2] The total number of genes in the pathway.
[3] The number of differentially expressed among those in the pathway.

Table S2. KEGG pathways identified via overrepresentation analysis of Colony A bees on 10th November 2025. None of the pathways was significant following a Bonferroni-Hochberg adjustment.

Regulation [1]	KEGG ID	KEGG Description	Genes in Category [2]	Differentially Expressed Genes [3]
Up	ame04977	Vitamin digestion and absoprtion	8	1
	ame04130	SNARE interactions in vesicular transport	17	1
	ame04624	Toll and Imd signaling pathway	27	1
Down	ame00981	Insect hormone biosynthesis	10	3
	ame04512	ECM-receptor interaction	13	1

[1] Up = more highly expressed in Poor Learners with high viral loads; Down = lower expression in Poor Learners with high viral loads. [2] The total number of genes in the pathway.
[3] The number of differentially expressed among those in the pathway.

Biology Open • Supplementary information

Table S3. Genes identified as potential targets of lncRNAs 102655375 in the Colony A group and as potential targets of lncRNAs 102654076, 113218947, and 107965291 in the Poor Learner group

Group	Regulator [1]	Targets OGSv3.2 ID [2]	OGSv1.x ID [3]	Gene ID [4]	Gene Description
Colony A	102655375	GB49322	GB14861	552711	anoctamin-4
		GB54313	GB13155	413386	uncharacterised protein coding
		GB54901, GB55397	GB13035	550961, *Vamp7*	vesicle-associated membrane protein 7
		GB41232	GB14096	551949	ovarian-specific serine/threonine-protein kinase Lok
		GB52719	GB11654	726124	segmentation protein Runt
		GB45874, GB45875	GB18913	724760	G-protein coupled receptor Mth2
		GB42418	GB15287	412372	pyruvate dehydrogenase phosphatase regulatory subunit, mitochondrial
		GB42552, GB42553	GB19513	100577098	enolase-phosphatase E1
		GB40064	GB12206	102655054	protein tyrosine phosphatase domain-containing protein 1
				102655080	long non-coding RNA
				104796166, *Mir9878*	microRNA 9878
	102654076	GB42828	GB14997	409085	guanine nucleotide exchange factor for Rab-3A
		GB55424, GB55425	GB18331, GB30192	725827, *InR-2*	insulin-like receptor-like
		GB40969		102655814	ras-related and estrogen-regulated growth inhibitor
		GB40686	GB19796	551426	uncharacterised protein coding
	113218947	GB45987		100578618	uncharacterised protein coding
Poor Learners	107965291	GB54319	GB13586	410052, Syt20	synaptotagmin 20
		GB49625, GB51241	GB11293	100578126	zinc finger protein 26-like
				102656274	long non-coding RNA

[1] The lncRNA purported to be serving as a regulator. [2] Gene IDs as specified in Official Gene Set (OGS) version 3. [3] Gene IDs as specified in Official Gene Set (OGS) version 1.x. [4] NCBI Gene ID and name (if available) (https://www.ncbi.nlm.nih.gov/gene/

Biology Open • Supplementary information

Table S4. Summary of Deformed Wing Virus (DWV) read counts from RNA-seq data, showing individual contributions from DWV-A recombinant and DWV-B lineages, their combined total, and the corresponding counts per million (CPM) relative to total sample reads. Alignments were performed using HISAT2 with stringent parameters to minimize cross-mapping (soft clipping disabled, increased mismatch/gap penalties, and a strict minimum alignment score). Only reads that mapped uniquely (i.e., with NH:i:1) and aligned for at least 50 bp were retained. CPM values were calculated as (DWV Total Reads/Total Reads) x 1,000,000. Mean CPMs are shown for Colony A and Colony B to compare viral load across colonies.

Sample	Colony	DWV-A recombinant	DWV-B	DWV (Total)	Total reads	DWV CPM
**1**	**B**	0	0	0	30900881.00	0
**2**	**B**	0	0	0	14192225.00	0
**3**	**A**	1	6	7	27836860.00	0.251465144
**4**	**A**	1	0	1	27799616.00	0.03597172
**5**	**A**	2	2	4	18822907.00	0.212507027
**6**	**A**	2	4	6	23549763.00	0.254779634
**7**	**B**	69	104	173	15565827.00	11.11408986
**9**	**B**	0	1	1	40902854.00	0.024448172
**10**	**B**	0	0	0	18358600.00	0
**11**	**A**	5,868	9,481	15,349	17843594.00	860.1966622
**12**	**A**	1,750,922	2,869,730	4,620,652	34879031.00	132476.5014
**13**	**B**	5	4	9	24571590.00	0.366276663
**14**	**B**	1,841,105	2,973,381	4,814,486	39056369.00	123270.1893
**15**	**A**	2	5	7	40882289.00	0.171223289
**16**	**A**	332,300	661,996	994,296	22324567.00	44538.19866
**17**	**B**	6	10	16	25825053.00	0.619553424
**18**	**A**	1	1	2	24133507.00	0.082872332
**19**	**A**	0	2	2	28736980.00	0.069596736
**20**	**B**	199,374	462,594	661,968	19314777.00	34272.61935
**21**	**A**	153,944	229,295	383,239	20975424.00	18270.85831
**22**	**B**	0	3	3	22525286.00	0.133183659
**23**	**A**	2,131	3,338	5,469	19529195.00	280.0422649
**24**	**B**	6	13	19	18237595.00	1.041804032
**25**	**B**	0	1	1	16099763.00	0.062112716
**26**	**A**	5	8	13	22681139.00	0.573163455
**27**	**A**	0	0	0	28175489.00	0
**28**	**B**	2	4	6	16475669.00	0.364173376
**29**	**A**	646,402	1,276,355	1,922,757	26005992.00	73935.1531
**30**	**B**	47	93	140	22259063.00	6.289572926
**31**	**A**	19	26	45	22533164.00	1.997056428
**32**	**B**	41	111	152	18653583.00	8.148568562
**33**	**B**	8	11	19	16129727.00	1.177949261
**34**	**B**	36	41	77	19393278.00	3.970447905
					**Mean CPMs**	
					Colony A	16897.78735
					Colony B	9269.184246

Biology Open • Supplementary information
